# Metabolic Profiling to Assess Response to Targeted and Immune Therapy in Melanoma

**DOI:** 10.3390/ijms25031725

**Published:** 2024-01-31

**Authors:** Chantale Farah, Lionel Mignion, Bénédicte F. Jordan

**Affiliations:** 1 Biomedical Magnetic Resonance Research Group, Louvain Drug Research Institute, Université Catholique de Louvain (UCLouvain), B-1200 Brussels, Belgium; chantale.farah@uclouvain.be; 2 Nuclear and Electron Spin Technologies (NEST) Platform, Louvain Drug Research Institute (LDRI), Université Catholique de Louvain (UCLouvain), B-1200 Brussels, Belgium; lionel.mignion@uclouvain.be

**Keywords:** melanoma, metabolic imaging, response to treatment, BRAF-targeted therapy, immune checkpoint inhibitors

## Abstract

There is currently no consensus to determine which advanced melanoma patients will benefit from targeted therapy, immunotherapy, or a combination of both, highlighting the critical need to identify early-response biomarkers to advanced melanoma therapy. The goal of this review is to provide scientific rationale to highlight the potential role of metabolic imaging to assess response to targeted and/or immune therapy in melanoma cancer. For that purpose, a brief overview of current melanoma treatments is provided. Then, current knowledge with respect to melanoma metabolism is described with an emphasis on major crosstalks between melanoma cell metabolism and signaling pathways involved in BRAF-targeted therapy as well as in immune checkpoint inhibition therapies. Finally, preclinical and clinical studies using metabolic imaging and/or profiling to assess response to melanoma treatment are summarized with a particular focus on PET (Positron Emission Tomography) imaging and ^13^C-MRS (Magnetic Resonance Spectroscopy) methods.

## 1. Introduction

There are two main types of skin cancer: non-melanoma skin cancer (NMSC), which includes squamous cell carcinoma (SCC) and basal cell carcinoma (BCC); and melanoma skin cancer. NMSCs are all curable by surgery whereas melanoma is the deadliest form of skin cancer with increased incidence rates [1,2]. If early detected melanomas are curable by surgery, the metastatic forms are highly refractory to treatments, with a five-year survival for only 25% of diagnosed patients which is due to major improvements these last decades [3]. Since the 1970s and the approval of the alkylating agent dacarbazine, the treatment of melanoma has indeed significantly evolved [4]. Overall response rates to dacarbazine were 10–20%, and a complete remission was observed in less than 5% of patients [5]. The discovery of a *BRAF* mutation, consisting of the substitution of V600E valine for glutamic acid at codon 600 that constitutively activates the MAPK (mitogen-activated protein kinase) pathway in more than 60% of melanomas, led to the use of BRAF inhibitors (BRAFi) targeting this mutation [6]. The first inhibitor approved by the FDA (Food and Drug Administration) for advanced melanoma patients was vemurafenib followed by dabrafenib and encorafenib [7,8]. Later on, combinations with MEK (mitogen-activated protein kinase kinase) inhibitors were approved to counteract resistance to BRAF inhibition [9,10]. In addition, over the last decade, immunotherapy with immune checkpoint inhibitors (ICI) constituted a second major breakthrough in the treatment of advanced melanoma patients [11]. To date, there is a lack of robust biomarkers to predict response to targeted or immunotherapy in melanoma.

### 1.1. Targeted Therapies in Melanoma

Vemurafenib was identified as the first selective inhibitor of the *BRAF* oncogene. It is an ATP-competitive inhibitor of *BRAFV600E*. It was approved by the FDA in 2011 [7] and was selected in different studies because of its better pharmacokinetic properties compared to other BRAF inhibitors [12]. Vemurafenib showed selectivity in *BRAFV600E*-mutant melanoma cells [13], and it displayed potent anti-tumor activity in preclinical melanoma models [14]. In the phase I BRIM (NCT00405587) study, a dose of 960 mg twice daily was established and well tolerated, the most frequent adverse events being skin rash, fatigue, nausea, and photosensitivity [15]. In a clinical study comparing vemurafenib to dacarbazine, it was shown that the median progression-free survival (PFS) was 5.3 months for the BRAF inhibitor (vemurafenib) in comparison with 1.6 months for dacarbazine [16]. Besides vemurafenib, two other BRAF inhibitors were approved for the treatment of advanced melanoma: dabrafenib, approved as monotherapy in 2013, and encorafenib, approved in 2018 [17]. Both inhibitors are ATP-competitive and selective. However, encorafenib has a much longer half-life (3.5 h) than vemurafenib (0.5 h) and dabrafenib (2 h), which make its inhibitory effects last longer [18]. If BRAF inhibitors were first approved for use as monotherapy, the combination of two molecules was recommended, due to the presence of intrinsic and acquired resistance mechanisms [19]. Indeed, only a small fraction of patients show durable complete response to BRAF-targeted therapy [20]. Several mechanisms of resistance were identified, including reactivation of the MAPK pathway [21], activation of substitutive pathways (PI3K or phosphatidylinositol 3-kinases—mTOR or mammalian target of rapamycin), *NRAS* and *MEK* mutations [22,23], *BRAF* splicing variants [24], *COT* (Cancer Osaka Thyroid Oncogene mitogen-activated protein kinase kinase kinase 8 or MEP3K8) alterations, *PTEN* (Phosphatase and TENsin homolog deleted on chromosome 10) loss, and *NF1* (Neurofibromatosis type 1) inactivation [25].

In this context, MEK was identified as a target to counteract resistance to BRAF inhibition, and trametinib was the first FDA-approved MEK inhibitor followed by cobimetinib and binimetinib [9,10]. MEK inhibitors are ATP non-competitive, highly selective, and reversible allosteric inhibitors [26]. Trametinib had first shown benefit as monotherapy in *BRAF*-mutant patients, leading to its FDA approval in 2013 [20]. In 2014, the combination with dabrafenib received approval [27] since it had shown a higher overall response rate (ORR) when compared to BRAFi as monotherapy (76% vs. 54%), with a progression-free survival of 12 months [10]. In 2015, a second MEK inhibitor, cobimetinib, received approval in combination with vemurafenib [9]. Later, a third MEK inhibitor, binimetinib, has been approved in combination with encorafenib [8], with a median overall survival (OS) of 33.6 months and a PFS of 14.9 months in comparison with an OS of 23.5 months for encorafenib alone [8,20]. The OS and PFS of BRAFi and MEKi based on clinical trials are illustrated in Table 1. If the combination of a BRAF inhibitor with a MEK inhibitor delayed the resistance, it did not prevent resistance due to secondary mutations in *MEK* [28] or due to alternative causes as listed above.

### 1.2. Immune Checkpoint Inhibitors in Melanoma

Immune checkpoint inhibition uses antibodies to inhibit the interaction between receptors expressed at the surface of activated T cells and on cancer cells to unleash immune responses [30]. CTLA-4 is a transmembrane glycoprotein; it is expressed on the surface of T cells [31]. CTLA-4 receptors are bound by the same ligands as CD28, but with a 20-times higher affinity, and thus they outcompete CD28 for ligands [32,33]. CTLA-4 activation interferes with T cell motility and ability to form stable interaction with antigen-presenting cells (APCs), thus decreasing the contact time between cells [34]. CTLA-4 are effective during the priming phase of naïve T cells and occur in the lymphatic tissue [35]. The interaction of CTLA-4 and its ligands leads to T cell inhibition. Using anti-CTLA-4 immune checkpoint blockade prevents this inhibition of T cell activation and promotes anti-tumor response [36]. In addition, the PD-1 receptor is expressed on the surface of activated T cells and was discovered in 1992 [37]. PD-1 ligands (PD-L1 and PD-L2) are expressed in many tissues and frequently in different cancer types, including melanoma [38]. PD-1/PD-L1 strongly counteracts T cell receptor (TCR) signal transduction even at very low PD-1 levels. As a result, PD-1 abrogates the production of cytokine and induces cell cycle arrest accompanied with a decrease in the pro-survival factor Bcl-XL [39]. Moreover, PD-1 causes a rapid switch from glycolysis to fatty acid B oxidation in CD8+ T cells and leads to reactive oxygen species accumulation, damage of mitochondria, and cell death. PD-1 signals are effective during the effector phase and occur within the peripheral tissues [35]. Therefore, the use of PD-1 blockade restores T cell functions [32].

In 2011, ipilimumab was the first FDA-approved antibody in melanoma inhibiting signaling via cytotoxic T-lymphocyte-associated proteins (CTLA-4) on T cells. Three years later, another anti-PD-1 (nivolumab) gained approval and an anti-programmed death-1 (anti-PD-1) monoclonal antibody, pembrolizumab, was approved for the treatment of metastatic melanoma patients. Of note, the adverse events were lower for pembrolizumab than for ipilimumab [40]. Like anti-PD-1, anti-PD-L1 antibodies (atezolizumab, avelumab, and durvalumab) have also shown promises in anti-tumor responses. The overall response rate of atezolizumab alone as monotherapy was consistent with the ORR of anti-PD-1 in melanoma patients (~30%) [41]. Finally, a combination of an anti-CTLA-4 with an anti-PD-L1 was shown effective: nivolumab alone or in combination with ipilimumab was associated with longer PFS and OS at 5 years than ipilimumab alone [42]. The OS and PFS of immune checkpoint inhibitors based on clinical trials are summarized in Table 2. Of note, immunotherapy was approved to treat people with advanced melanoma regardless of whether their tumors had *BRAF* mutation or not [43]. In contrast to targeted therapies, immunotherapy with ICI also shows more durable clinical responses, lasting for several years. However, primary resistance mechanisms are still present [44].

### 1.3. Combination of Targeted Agents with Immunotherapy

Targeted and immune therapy have changed the management of advanced stage melanoma. Both have improved the outcomes of patients with melanoma. However, some limitations and toxicities are still present [46]. Combination studies were considered to counteract the limitations of both therapies, i.e., the lack of durability of response for targeted therapies and the lower initial response rate for immune therapy. Besides the limitations, there is evidence that targeting the MAPK pathway modulates anti-tumor immunity via several mechanisms [46]. First, it inhibits the suppressive immune cells such as myeloid-derived suppressor cells (MDSCs) and regulatory T cells (Tregs) [47]. Second, during treatment with BRAFi there was an increase in the expression of tumor-associated antigens that can promote recognition of tumor cells by T lymphocytes. Finally, the higher MHC (major histocompatibility complex) expression was associated with MAPK inhibition and this promotes T cell activation and facilitates T cell anti-tumor response [47]. Understanding these immune effects helps optimize the use of these therapies alone or in combination.

The first clinical study that combined targeted therapy (vemurafenib) with an immune checkpoint inhibitor (anti-CTLA-4 ipilimumab) showed severe hepatic toxicity and the researchers ended up stopping the trial. By contrast, ipilimumab combined with dabrafenib was better tolerated whereas the triple combination of ipilimumab, dabrafenib, and trametinib was also associated with gastrointestinal toxicities [48,49]. Later on, clinical trials combining targeted and immunotherapy shifted towards anti-PD-1/anti-PD-L1 agents [50]. In this context, multicenter studies assessed the safety of triple combinations BRAFi/MEKi/Anti-PD-1. While the ORR in the triple combinations (KEYNOTE-022, IMSPIRE150, and COMBI-i) was between 63% and 69% (Table 3), the studies reported severe grade 3 or 4 adverse events (AEs) compared to double combinations. Interestingly, combining anti-PD-1 with MEKi was associated with a better disease control, with less toxicity than the triple combination, supporting the need for further studies [50].

Combined immunotherapy with targeted therapy versus sequential immunotherapy/targeted therapy needs to be considered and requires an oncologist to decide which treatment option is necessary. It remains a major therapeutic challenge to decide which treatment will induce the best efficacy as a first-line treatment [53]. Sequencing anti-PD-1 treatment before targeted therapy influences the response in *BRAFV600E*-mutant melanoma [54]. Hence, most patients who have received anti-PD-1 after BRAF/MEK inhibition required hospitalization for adverse events and dose interruptions [55]. This may be due to micro-environmental features associated with immunotherapy resistance that promote cross-resistance to targeted therapies [56]. Another sequential combination trial (DREAMseq) was conducted recently in 2022 to determine the optimal treatment sequence and has shown that the combination of nivolumab/ipilimumab followed by targeted therapy is preferred in most patients [57].

Decision-making criteria are increasingly complex regarding whether to choose targeted therapy or immunotherapy as a first-line treatment [58]. In this context, predictive biomarkers that aim to identify patients and predict responders or non-responders are needed and have been included in trials as secondary endpoints [53]. Several biomarkers have been studied in metastatic melanoma such as clinical endpoints (tumor burden and metastatic sites), blood markers (serum lactate dehydrogenase (LDH), neutrophils, monocytes, and lymphocytes levels), stool (gut microbiome), tumor tissue markers (mutational analysis, tumor infiltrating lymphocytes, PD-L1, interferon-γ), and imaging biomarkers [59]. However, there is to date still a lack of clinically validated robust biomarkers. In parallel, metabolic plasticity has been shown to be responsible for treatment resistance in several cancers [60]. Within this scope, recent studies suggest that metabolic shifts observed in response to melanoma therapy could be linked with sensitivity or resistance to targeted or immune therapy, and are described in the next section. Imaging these metabolic shifts could therefore be useful to assess/predict the response to treatment in melanoma.

## 2. Aberrant Metabolism in Melanoma

Deregulated metabolism is one of the common hallmarks of cancer [61]. Tumors are heterogenous and this heterogeneity creates a complex metabolic pattern. The majority of proliferating melanoma cells use glycolysis to quickly produce ATP to sustain their proliferation and provide macromolecules for cell division [62]. In vitro and in vivo, resistance to BRAF inhibitors induces different metabolic changes including decreased glycolysis [63,64], dependence on glutamine metabolism [65,66,67], increased serine biosynthesis [68,69], and activated mitochondrial oxidative signature [65,70].

*BRAF*-mutated melanomas exhibit increased glycolysis [71,72]. This dependence on glycolysis sensitizes *BRAF*-driven melanomas to BRAF inhibitors [73]. Following treatment with BRAFi, a decreased glycolysis and increased oxidative phosphorylation were observed in these tumors [74,75]. Moreover, BRAFi induced a decrease in the uptake of glucose using ^18^F-FDG (fluorodeoxyglucose) in melanoma xenografts and patients [63,64].

Besides glucose, glutamine, the most abundant amino acid circulating in the bloodstream [76], is another important fuel for cancer cells [77]. It has a major role in producing ATP and replenishing TCA (tricarboxylic acid) cycle intermediates, and can be synthesized in skeletal muscles, adipose tissue, and lungs. It is a non-essential amino acid under physiological conditions that becomes essential under pathological conditions [76]. Following cellular transport through different transporters such as NAD+ alanine serine cysteine transporter 2: SLCA5 (LAT1) and SLC1A5 (or ASCT2), glutamine is hydrolyzed into glutamate by glutaminases (GLS); different enzymes including glutamate dehydrogenase mediate the deamination of glutamate to alpha-ketoglutarate [78]. Glutamine dependency has been shown in melanoma cell lines, thus demonstrating that glutamine is an essential amino acid as much as glucose [79]. In this context, c-Myc has been shown to be a target of ERK and enhances glutamine uptake and glutaminolysis by regulating the transcription of genes coding for SLCA5 and GLS, respectively [80,81]. Studies have shown the glutamine dependency of A375, SKMEL5, and G361 melanoma-resistant cells to BRAF inhibitors [67].

Finally, glycolysis produces ATP and intermediates that support the PPP (Pentose Phosphate Pathway) and the serine/glycine pathway in melanoma cells. Phosphoglycerate dehydrogenase (PHGDH) catalyzes the first step of the serine/glycine pathway and is overexpressed in melanoma cell lines and tumors [82]. Serine has a major role in the synthesis of purine and pyrimidine. It can be synthesized from 3-phosphoglycerate and converted to glycine by serine hydroxymethyltransferase (SHMT) [83]. Several PHGDH inhibitors have been developed. One study has shown that targeting PHGDH resensitizes resistant melanoma to MAPK inhibition [69,84]. Besides serine, melanoma cancer cells can also rely on other non-essential amino acids such as aspartate and glycine to meet their bioenergetics needs. Aspartate represents a limiting metabolite for cancer proliferation under hypoxic conditions [85], while high rates of glycine consumption are associated with increased cell proliferation [86].

A metabolic symbiosis was identified between tumor cells based on lactate exchange [87]. Due to the heterogeneity within the tumor, there are two types of tumor populations: the oxidative and the hypoxic tumor cells. The hypoxic tumor cells rely on glycolysis. The lactic acid is released by these cells through the MCT4 and is used by oxidative cancer cells, which makes glucose available for hypoxic cells [88]. The oxygenated tumor cells use lactate, which is oxidized to pyruvate by LDH1 and fuels the TCA cycle to produce energy. This metabolic preference and cooperation are the core of the tumor cell survival under hypoxia. In addition, this metabolic crosstalk has been also documented between cancer cells and cancer-associated fibroblasts [89]. A metabolic symbiosis has been shown to be vital for melanoma progression [90].

### 2.1. Crosstalks between MAPK Signaling and Melanoma Cell Metabolism

**Figure 1:**
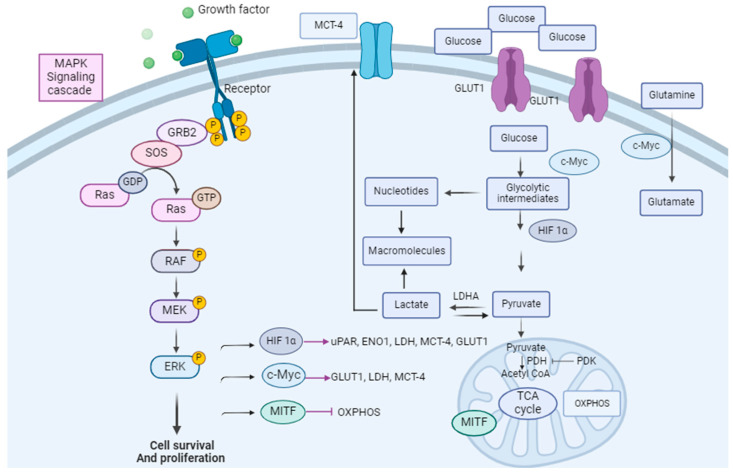
Link between the MAPK pathway and melanoma cell metabolism. The MAPK pathway is linked to tumor cell metabolism via several factors: HIF-1α, c-Myc, and MITF. These are direct targets for p-ERK. They are modulators of glycolysis and glutaminolysis. Adapted from [115].

In melanomas, the Warburg phenotype is driven by the activation of signaling pathways, particularly the MAPK pathway. Glucose metabolism is regulated by oncogenic *BRAF* via several factors: avian myelocytomatosis viral oncogene homolog (c-Myc), hypoxia-inducible factor 1 alpha (HIF-1 alpha), and microphtalmia-associated transcription factor (MITF), which are direct targets for phospho-ERK (p-ERK) [91,92] (Figure 1).

c-Myc is a proto-oncogene that has a pivotal role in cell proliferation, differentiation, and apoptosis. Its overexpression sensitizes cells to apoptosis [93]. c-Myc overexpression drives melanoma metastasis [94]. The ERK-mediated phosphorylation of Serine 62 results in the stabilization of c-Myc while the phosphorylation at threonine 58 by the glycogen kinase synthase (GSK-3β) targets c-Myc for proteasomal degradation [95]. Among thousands of c-Myc targets, which have a role in protein biosynthesis, cell metabolism, and the cell cycle, the glycolytic genes *GLUT1*, *MCT1*, *MCT4*, *GAPDH*, and *LDHA* are direct targets relevant to the tumor metabolic phenotype [96,97,98].

HIF-1 alpha regulates the transcription of genes encoding for glucose transporters (GLUT1/2), hexokinase I/II, monocarboxylate transporters (MCT4), and pyruvate dehydrogenase kinase 1 (PDK1). For instance, HIF-1 alpha activates the pyruvate dehydrogenase kinase (PDK), which is a PDH inhibitor, preventing the entrance of pyruvate into the TCA cycle [99,100], resulting in an increased glycolytic flux and a decreased mitochondrial respiration [79,101,102]. In addition, HIF-1 alpha increases the expression of the urokinase plasminogen activator receptor (uPAR) which is expressed in one-third of melanoma cases [103,104]. The uPAR connection with EGFR (Epidermal Growth Factor Receptor) and the PI3K/mTOR/HIF pathway drives a glycolytic phenotype in melanoma [105]. In addition, uPAR regulates the expression of Enolase-1 (ENO1) and extracellular matrix metalloproteinase (EMPPRIN), which both contribute to the invasive phenotype of melanoma metabolism and glycolysis [105]. Specifically, HIF-1 alpha and c-Myc upregulate MCT4 and lead to lactate secretion into the tumor micro-environment. Monocarboxylate transporters (MCTs) are the main bidirectional transporters of lactate. It has been shown that the expression of *GLUT1* and *MCT4* is increased from primary to metastatic melanoma tumors [106]. Finally, MITF inhibition via the MAPK pathway has also been shown to promote glycolysis [91].

Accordingly, targeting melanoma metabolism to overcome resistance has been studied in preclinical trials. To date, there is still no approved drug modulating melanoma metabolism to treat patients with melanoma. Within this scope, many studies have explored the role of glucose and glutamine metabolism and the rationale to target it for cancer treatment. For instance, one ongoing clinical trial is currently testing the combination of CB-839 (glutaminase inhibitor) with the immune checkpoint inhibitor nivolumab in melanoma patients [107,108]. Moreover, biguanides (anti-diabetic drugs) such as metformin and phenformin have demonstrated anti-tumor activity [109]. It is known that adenosine monophosphate activated protein kinase (AMPK) is activated by metformin and phenformin. In addition, it has been shown that the oncogenic *BRAFV600E* suppresses the activity of AMPK, providing the rationale of combining BRAFi with metformin and phenformin for the treatment of melanoma [110,111,112]. The use of metformin has shown no clinical benefit on melanoma patients who progressed with targeted or chemotherapy [113]. Nonetheless, in some cases metformin accelerates tumor growth in vivo in resistant *BRAF*-mutant melanoma cells [114]. Clinical trials have been performed to test metformin treatment against melanoma: in particular, the combination of BRAFi/MEKi or anti-PD-1 or chemotherapy with metformin, NCT01638676, NCT02143050, NCT01638676, NCT02190838. Phenformin showed more potent anti-tumor effects when compared to metformin. However, it also showed serious side effects in diabetic patients, which led to its withdrawal.

### 2.2. Crosstalks between Immune Cell Signaling and Melanoma Cell Metabolism

**Figure 2:**
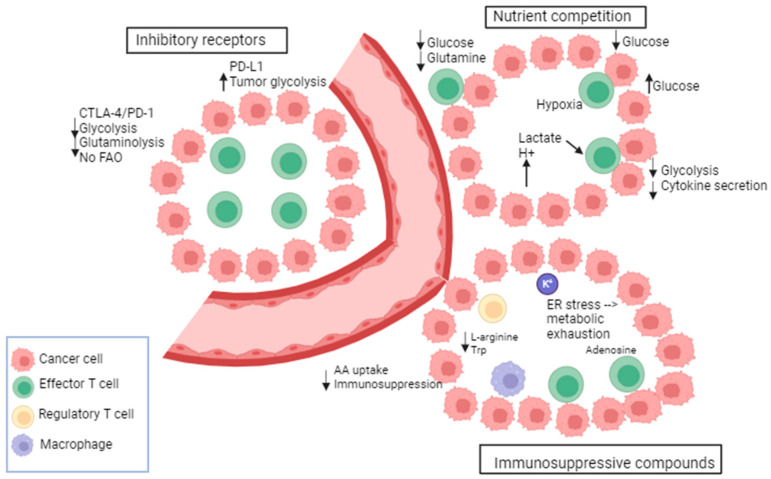
Metabolic and immunological checkpoints that impede T-cell-mediated immunity. Tumors adjust their metabolism in response to nutritional stress to compete for glucose and amino acids (AA), thereby suppressing T cells. Immune checkpoint receptors exert negative metabolic functions in T cells. Tumor metabolites impact the function of T cells. Byproducts of immunosuppressive immune cells contribute to the metabolic exhaustion of T cells. Adapted from [117].

Metabolic transitions are not exclusive to cancer cells; they are also observed in other proliferating cell types including activated T cells and others [116]. T cell bioenergetic is influenced by several mechanisms including the following: nutrient competition, immunosuppressive compounds, and immune checkpoints [117] (Figure 2). Recent studies indicate a notable difference in energy consumption between active and resting immune cells. For instance, the metabolic activity of naïve T cells remains largely constant with minimal proliferation. Consequently, they require only essential nutrients and a minimal rate of glycolysis and biosynthesis to sustain their functions. The primary source of ATP in these cells is OXPHOS [118]. Once stimulated by external triggers, T cells undergo a metabolic shift characterized by high nutrient uptake, elevated glycolysis, increased synthesis, and the accumulation of proteins, lipids, and nucleotides [118]. Also, effector immune cells, like activated cytotoxic T cells, undergo metabolic reprogramming in order to carry out various functions, such as eliminating cancer cells and releasing cytokines [119].

A metabolic and nutrient competition exists between tumor and immune cells in the tumor micro-environment (TME) as the energy demand is high in the TME [120]. This creates a continuous battle for nutrients. The glucose metabolism of cancer and immune cells in the TME was shown to be mediated by the glucose transporters GLUT1 and 3. A reciprocal change in the glucose uptake was observed between immune cells and cancer cells. For instance, the GLUT1-mediated cancer cell uptake is suppressed when the GLUT3-mediated immune cell uptake is increased [121]. In this context, CD28 promotes the upregulation of GLUT1 and allows glucose uptake [122]. It has been demonstrated that in TCR-stimulated T cells, the glucose uptake was increased upon the induction of CD28 co-stimulation via Ab-coated beads [123]. Moreover, the cytolytic activity of T cells and the IFN-gamma (interferon-gamma) production are regulated by glucose consumption [124]. Further, the oxygen consumption by oxidative melanoma cells leads to deprivation of oxygen from T cells, decreased anti-tumor immunity, and poor response to PD-1 blockade. In this context, the inhibition of oxidative metabolism showed an increased sensitivity to PD-1 blockade [125]. There is evidence that tumors with gain-of-function mutations in glycolytic enzymes exhibit high resistance to T-cell-mediated immunity. For instance, in renal cell carcinoma, the high expression of glucose transporter GLUT1 is correlated with low CD8+ T cell infiltration in the tumor [126]. Indeed, several investigations have provided evidence that the glycolytic activity of cancer cells can hinder the uptake of glucose by tumor-infiltrating lymphocytes, leading to T cell exhaustion, an evasion of the immune response [127].

Besides nutrient competition, many byproducts of cellular metabolism have an immunosuppressive role in the TME. As per the findings, glycolytic metabolites such as lactate have been shown to have a detrimental impact on the immune function by modulating the activation and maturation of dendritic cells [128,129], impairing T cell proliferation and cytokine production [130]. Furthermore, elevated levels of lactate and reactive oxygen species (ROS) are commonly observed in the TME and represent other mechanisms for immune cell suppression. These factors collectively contribute to cancer progression and facilitate immune evasion [129]. In addition, the accumulation of K+ in the interstitial fluid of the tumor acts as a suppressor of amino acid and glucose transporters in T cells. An additional immunosuppressive way involves the adenosine produced by suppressive Treg cells which binds to adenosine receptors (A2AR) on cytotoxic T cells and suppresses their function through the reduction of NF-kB (nuclear factor-kB) signaling [131]. Furthermore, tumor-derived cholesterol can induce metabolic and ER stress in T cells preventing cytokine production by T cells and leading to increased immunosuppressive molecule expression (PD-1, TIM-3, or T cell immunoglobulin and mucin domain molecule 3, LAG-3, or lymphocyte activation gene-3) [132].

In addition to tumor cells, other immune cells consume nutrients that are beneficial to T cells and thus produce immunosuppressive metabolites. For instance, M2 macrophages utilize L-arginine through the upregulation of arginase-1 leading to the depletion of arginine required for protein synthesis in T cells. Moreover, indoleamine 2,3 dioxygenase (IDO) is highly expressed in M2; it metabolizes tryptophan into kynurenine, thus depleting tryptophan availability for T cells. Finally, the inhibitory receptors (IRs) such as PD-1, CTLA-4, LAG-3, and TIM-3 are a defining characteristic of T cell dysfunction. These IRs impair T cell response against tumors by altering their metabolism. PD-1 alters T cell function by increasing CPT1a, a fatty acid oxidation (FAO) enzyme, and by decreasing glycolysis. In contrast, PD-L1 expression in tumor cells enhances glycolysis and deprives T cells of glucose [133]. There is also evidence that the MAPK inhibition influences PD-L1 expression. MAPK activation in melanoma cells that are resistant to BRAF inhibitors promotes the expression of PD-L1; however, MEKi combinations showed a downregulation of MAPK and a suppression of PD-L1 expression [134]. Furthermore, it has been shown that hypoxia induces PD-L1 expression and activates Hedgehog (Hh) signaling [135]. Of note, Hh is a pathway that has a role in growth and in different tissues during embryonic development [136]. Moreover, PD-L1 is a direct target of HIF-1α in myeloid-derived suppressor cells (MDSC). PD-L1 inhibition under hypoxia enhanced T cell proliferation. In this context, tumor metabolism was altered following PD-L1 blockade [127]. Other IRs such as CTLA-4 inhibit the function of T cells and downregulate the glutamine transporters and GLUT1, thus decreasing the bioenergetic potential of T cells [137]. LAG-3 also impairs T cell activation and proliferation.

## 3. Imaging Metabolism as a Marker of Response to Treatment in Melanoma

In oncology, detecting treatment response at an early stage is crucial. This can allow for treatment adjustments, such as intensifying therapy or discontinuing ineffective treatment to prevent unnecessary side effects. In treatment monitoring, the measurement of tumor size remains the most used method for evaluating tumor response. The World Health Organization (WHO) suggested the initial guidelines in 1979. They were later revised into the Response Criteria in Solid Tumors (RECIST) in the 1990s, focusing on the maximum diameter of the tumor. However, in some cases the reduction in tumor size occurs at later stages of treatment. In addition, targeted therapies can result in tumor size stabilization rather than in a significant reduction in size, still with significant increase in overall survival. Therefore, it is crucial to use non-invasive imaging to evaluate early response to treatment. Indeed, the parameters of the TME, including the metabolic phenotype or plasticity, can be affected by a therapy earlier than the tumor size is affected, due to the existing links between tumor cell signaling and metabolic pathways. This is even more significant to assessing the response to immunotherapy, where the TME plays a critical role. Several non-invasive imaging modalities exploit the perturbed metabolic state of tumors. Those methods have the potential to assess tumor response by assessing metabolic shifts or plasticity in response to cancer treatment, and thereby can provide information for treatment monitoring and potentially predicting survival for cancer patients.

### 3.1. PET (Positron Emission Tomography) Imaging to Assess Response in Melanoma

Among various non-invasive molecular imaging methods used in both preclinical and clinical settings, positron emission tomography (PET) has emerged as a powerful tool for cancer detection and monitoring [138]. The PET detection method is based on the use of a radiolabeled substrate that is a short-lived positron-emitting isotope such as ^18^F. The positron combines with an electron, and the two opposite charges annihilate each other and produce two gamma rays (annihilation photons) of each 511 keV emitted in opposite directions at approximately 180° from each other, which are detected by small detectors that are placed in adjacent rings around the patient [138]. In cancer, the method is based on the high glucose consumption in tumors with respect to most healthy tissues, and therefore uses the glucose analog 2-deoxy-2-[fluorine-18] fluoro D-glucose (^18^FDG) as a tracer of the glucose uptake into cells by glucose transporters [138]. Of note, the ^18^FDG tracer is not metabolized further inside the cells (i.e., it is not phosphorylated by the hexokinase enzyme into G6P (glucose-6-phosphate)), contrarily to glucose. Notably, a reduction in ^18^FDG uptake has been observed in melanoma cells in laboratory studies as well as in human tumors following targeted therapy [63,139]. More specifically, the inhibition of BRAF has been found to result in decreased ^18^FDG uptake in both *BRAF*-mutated melanomas in xenograft models and in patients [63,139]. In particular, early ^18^FDG-PET scans at day 15 in patients treated with targeted therapy (dabrafenib) on phase I trials demonstrated early, complete, or partial metabolic responses, potentially with prognostic significance [64,139]. However, heterogeneity of response was common in metastatic melanoma treated with dabrafenib [63,139].

Studies have also investigated the use of ^18^FDG-PET as an early predictor of immune checkpoint blockades in patients with melanoma [140,141]. As an example, ^18^FDG-PET performed after two cycles of ipilimumab has demonstrated high predictability for the final treatment outcome in patients with progressive and stable metabolic disease in metastatic melanoma. However, the distinction of patients between pseudo-progression and progressive disease has been shown in several consecutive studies to be limited using ^18^F-FDG-PET/CT, consisting of the combination of PET imaging with computed tomography (CT), providing anatomical information simultaneously with the ^18^FDG uptake maps [142,143]. All studies assessing response to immunotherapy with ^18^FDG-PET along with associated standardized evaluation criteria were recently reviewed by Mangas Losada et al. [141]. Importantly, in 2018, standardized criteria, PERCIMT (PET Response Evaluation Criteria for Immunotherapy), were defined for melanoma, including morphologic and metabolic response to assess the clinical benefit of immunotherapy [141,144,145]. In parallel, standard PERCIST (Positron Emission tomography Response Criteria in Solid Tumors) criteria were also revisited in the scope of immunotherapy, including iPERCIST [146] and immunotherapy-modified PERCIST5 (imPERCIST) [147]. Although these criteria are neither globally accepted nor clearly established yet, they consider the fact that immunotherapy can cause non-conventional patterns of response and must be considered for personalized evaluation [141]. Despite the efforts that have been made to standardize the criteria to assess response to immunotherapy with ^18^FDG-PET, many challenges remain in the field, including the distinction between atypical response patterns, including pseudo-progressive disease, hyper-progressive disease, dissociated response, and sustained response [145]. In the future, radiomic features from PET/CT could be useful to better predict response to immunotherapy in melanoma [148].

There is therefore room for improvement in melanoma response monitoring using metabolic imaging. As ^18^FDG-PET only probes glucose uptake, methods able to assess glucose downstream metabolites or real-time metabolic fluxes are of interest and will be described in the next section.

### 3.2. Magnetic Resonance Metabolic Profiling to Assess Response to Melanoma Therapy

^13^C-metabolic tracing/fluxomic involves introducing a nutrient labeled with a stable (i.e., non-radioactive) isotope that can be recognized by nuclear magnetic resonance (NMR) spectroscopy or mass spectrometry (MS) [149]. As most of the carbon in the human body is in the form of the carbon-12 (^12^C) isotope, the signal from naturally abundant ^13^C is very low [149], which constitutes an advantage in tracing experiments where the goal is to monitor the metabolic conversions of an exogenous ^13^C-labeled substrate with a very low background signal. In this context, ^13^C is commonly used to track carbon fates from ^13^C-glucose, and the transfer of the isotope label to downstream metabolites occurs as a result of metabolic activity in tissues [149]. Metabolomics and stable isotope tracing provide complementary views of the metabolic phenotype, with metabolomics providing a broad view of steady-state metabolite abundance whereas isotope tracing adds a component of dynamic metabolite turnover [150]. With respect to the detection method, both NMR and mass spectrometry are being used. NMR spectroscopy measures the transition from one stage of energy to another of nuclei possessing a spin ≠ 0 (i.e., atoms with an odd mass number or atoms with a peer mass number and an odd proton number), whereas MS determines the mass of a molecule by assessing the mass-to-charge ratio *m*/*z* of its ion, which is generated by inducing either the loss or gain of a charge from a neutral species. With respect to MS, extracted metabolites are often separated using gas chromatography (GC-MS), liquid chromatography (LC-MS), and capillary electrophoresis (CE-MS). Whereas NMR and MS methods have often been compared in terms of sensitivity, which is largely in favor of MS, the complementarity between both techniques is strong, with MS providing sensitive information on the fractional enrichment of mass isotopomers and NMR providing detailed positional information on isotope enrichments (i.e., a more reliable metabolite structure identification) [150]. The technique is being considered to assess sensitivity to therapy in the preclinical setting as well as in clinical studies, with a higher specificity than ^18^F-FDG-PET, by monitoring the fate of glucose beyond glucose uptake by the tumor cells. The clinical safety in humans has already been demonstrated, as the group of Deberardinis [151] initially developed a protocol to analyze human tumor metabolism through the IV infusion of ^13^C-glucose in patients, and ex vivo detection of the downstream metabolites on tumor biopsies or on resected tumors [151]. The method has been applied to several tumor types in patients [75,152,153] and in mouse xenografts [154], yet with the disadvantage of an ex vivo detection, which is unpractical for longitudinal monitoring.

Besides ex vivo ^13^C-metabolic tracing, hyperpolarized (HP) ^13^C-MRI is an emerging molecular imaging method that allows for the rapid, non-invasive, and pathway-specific investigation of dynamic metabolic and physiologic processes that were previously inaccessible to imaging [155]. The technique overcomes the low intrinsic sensitivity of ^13^C magnetic resonance spectroscopy (MRS) that hampers in vivo studies because of long acquisition times. Indeed, one method for overcoming the low signal of ^13^C-MRI is through the external hyperpolarization and injection of exogenous ^13^C-labeled molecules, thereby enabling the imaging of central metabolic pathways such as glycolysis and the tricarboxylic acid cycle in real-time [156]. Hyperpolarization refers to a temporary increase in the proportion of nuclear spins aligned with the main magnetic field, thereby temporarily boosting the difference in the spin populations of the two energy states, which is directly linked to the sensitivity of NMR [156]. Several methods can be used to achieve hyperpolarization including dynamic nuclear polarization (DNP) and parahydrogen-induced polarization (PHIP) [156]. By this way, dynamic metabolic processes can be assessed in vivo by temporarily boosting the ^13^C-NMR signal of some key metabolic substrates allowing the in vivo dynamic measurement of metabolic conversions [156,157,158]. The technique presents the unique advantage of probing real-time metabolic fluxes in vivo and therefore does not require excising the tumor sample. In particular, HP ^13^C-MRS can image the real-time conversion of HP-[1-^13^C]pyruvate to [1-^13^C]lactate, catalyzed by LDH, and is a promising preclinical and clinical imaging biomarker of aggressiveness and early treatment response [159,160,161]. The technique measures the ^13^C-lactate-to-^13^C-pyruvate ratio, reflecting the activity of LDH. The ratio is also influenced by the activity of monocarboxylate transporters, including MCT1 [162] and by the level of hypoxia [163]. Potential clinical applications of hyperpolarized ^13^C-MRI in oncology include using metabolism to stratify tumors by grade, selecting therapeutic pathways based on tumor metabolic profiles, and detecting early treatment response through the imaging of shifts in metabolism that precede tumor structural changes, as recently reviewed [156]. The first human study with HP-[1-^13^C]pyruvate demonstrated the safety of the method, along with the evidence that ^13^C-MRI could identify a prostate tumor that was not visible with conventional ^1^H-MRI [164]. This was followed by numerous clinical studies with HP-[1-^13^C]pyruvate in pancreatic, kidney, breast, and brain cancers [165,166]. The method has further been applied for tumor grading, showing higher levels of pyruvate–lactate exchange in more aggressive tumors [163,167,168]. With respect to therapy monitoring, studies identified early response to chemotherapy or targeted therapy in several human cancers [169,170,171] and in numerous preclinical models, including breast, pancreatic, hepatocellular, glioblastoma, lymphoma, prostate, head and neck, ovarian, and cervical cancer xenografts, as recently reviewed [156].

Although several works point out the role of glucose metabolism in melanoma cell signaling, as summarized in Section 2.1 and Section 2.2 of this review, little has been done so far using ^13^C-metabolic tracing/fluxomic or ^13^C real-time metabolic imaging with respect to the assessment of response to advanced melanoma therapy. In vitro works suggested that, following treatment with BRAF inhibitors, mutant *BRAF* human cancer cells (WM266.4 and SKMEL28) showed an inhibition of HP ^13^C-pyruvate–lactate exchange, associated with depletion in hexokinase 2 and MCT1 and MCT4 [74]. Similarly, our group showed that BRAF inhibitors in human A375-sensitive melanoma cells impaired glycolysis in vitro, as attested by a decreased ^13^C-pyruvate–^13^C-lactate exchange in response to vemurafenib [172]. However, in human A375 melanoma xenografts in vivo, the HP pyruvate–lactate exchange was increased after BRAF inhibition. This paradoxical effect suggested a significant influence of the tumor micro-environment on the tumor metabolic phenotype. Therefore, we further characterized the ^13^C-metabolic profile in response to BRAF/MEK-targeted therapies in YUMM1.7 [173] and YUMMER1.7 [174] syngeneic melanoma xenografts characterized for *BRAFV600E*/wt *PTEN*−/− *CDKN*2−/−, allowing the use of immunocompetent mice to fully integrate all aspects of the tumor micro-environment. We evaluated the relevance of HP ^13^C-pyruvate as well as of ^13^C-MRS fluxomic after [U-^13^C]-glucose injection, as potential markers of response to targeted and immune therapies in the preclinical setting [175,176].

Regarding targeted therapies, ^13^C-MRS fluxohimic was able to assess response in YUMM1.7 xenografts. In particular, a significant decrease in ^13^C-lactate production after ^13^C-glucose injection was observed as soon as 24 h after treatment initiation with a single BRAF inhibitor (vemurafenib), with a single MEK inhibitor (trametinib), and with the combined BRAF/MEK inhibitors (vemurafenib + trametinib), contrarily to the control group. This suggests that ^13^C-glucose could be a marker of response to melanoma targeted therapy since all treatments were proven efficient to delay the growth of the YUMM1.7 xenografts. However, the technique was not able to reflect the higher efficacy of the combined targeted therapies in comparison with the single inhibitors in terms of growth delay. On the contrary, HP ^13^C-pyruvate could discriminate positive response in four out of five tumors treated with the combined BRAF/MEK inhibition, in comparison with the single inhibitors, showing potential for a better sensitivity than ^13^C-fluxomic when used for individual monitoring, i.e., by comparing the ^13^C-lactate-to-^13^C-pyruvate ratio before and 24 h after treatment for each individual tumor [175].

With respect to immune checkpoint inhibitors, we characterized the ^13^C-metabolic profile in response to PD-1 blockade in YUMMER1.7 syngeneic melanoma xenografts, described to be sensitive to immunotherapy [176]. A significant decrease in the ratio of HP ^13^C-lactate to ^13^C-pyruvate was observed in vivo after one cycle of immunotherapy, which was not observed in the isotype control group, and that was in accordance with a decrease in LDH-A expression in the same tumors. However, a lack of change in ^13^C-lactate production was observed using ^13^C-glucose tracing at the same timepoint, illustrating that steady-state metabolite concentrations do not necessarily reflect the activity of a metabolic pathway, contrarily to real-time metabolic measurements. Importantly, the change in the ^13^C-lactate-to-^13^C-pyruvate ratio preceded any significant change in tumor volume in the anti-PD-1 and isotype groups that were significantly different after two cycles of immune therapy. Of note, at this timepoint, T cell and macrophage populations showed a trend to increase in the peripheric regions of the treated tumors. The interplay between the anti-PD-1 effect and the pyruvate-to-lactate conversion is potentially the consequence of several mechanisms. In particular, it was reported that PD-1 expression is regulated by the lactate levels present in the TME, and that the metabolic enzyme LDH-A plays a crucial role in the modulation of immune anti-tumoral responses to anti-PD-1 [177]. Accordingly, it was recently shown that the blockade of LDH-A improves the efficacy of anti-PD-1 therapy in B16 melanoma [177]. These data illustrate the potential of non-invasive real-time monitoring of the metabolic flux to assess response to immunotherapy before any classic (RECIST) clinical criteria are affected.

Interestingly, Zhang et al. [178] demonstrated recently using stable isotope tracer-based metabolic flux studies that immunologically hot melanoma utilizes more glutamine than immunologically cold melanoma in vivo and in vitro. This was corroborated by analyses of human melanoma RNA-seq data, demonstrating that glutamine transporters and other anaplerotic gene expression positively correlated with lymphocyte infiltration and function [178]. In addition, it has been shown that tumors induced by overexpression of c-Myc have increased glutamate production following ^13^C-glutamine injection, suggesting that glutamine metabolic changes may be the result of an overexpression of c-Myc [179]. It would therefore be relevant to consider ^13^C-glutamine tracing experiments in the YUMMER1.7 xenografts in response to anti-PD-1 immunotherapy.

## 4. Discussion

Preclinical data suggest that metabolic imaging approaches could be assessed in the clinical setting in order to identify advanced melanoma patients who are more likely to respond to targeted, immune, or combined therapies. Additionally, it may contribute to the development of more effective treatment strategies for advanced melanoma patients by enabling early monitoring of treatment response and potentially predicting the emergence of drug resistance in the transition towards personalized medicine. In particular, this could help in the determination of the sequential administration of targeted and immune therapy on an individual basis. In this context, hyperpolarized ^13^C-pyruvate MRI has been successfully translated into the clinical setting, with initial safety studies, followed by studies correlating the lactate-to-pyruvate ratio with the tumor grade in several cancers and for detection of early treatment response [156]. Clinical research is ongoing to evaluate the accuracy of the method for monitoring response to new anti-cancer drugs, yet with the need for new technical developments to enable the use in a routine clinical setting [156]. In addition, the combination with emerging non-invasive metabolic imaging methods could be of interest to assess response to melanoma therapy, including deuterium (^2^H) metabolic imaging (DMI), combining deuterium magnetic resonance spectroscopic imaging with oral intake or intravenous infusion of ^2^H-labeled substrates (such as deuterated glucose) to generate metabolic maps in vivo [180,181]. Similar to ^13^C-glucose, DMI using ^2^H-glucose can reveal glucose metabolism beyond mere uptake as it undergoes further downstream metabolism and can be detected via high concentration metabolites such as ^2^H-lactate and ^2^H-glutamate/glutamine in vivo, representing the distinct fates of glucose [182]. Importantly, DMI has directly shown clear translational potential in studies with human subjects [183], yet with the need for ^2^H-^1^H MR clinical coils. Finally, the complementarity of the emerging metabolic imaging methods with ^18^FDG-PET should be assessed in future clinical studies.

## Figures and Tables

**Table 1:** PFS and OS of combined BRAF and MEK inhibitors.

Clinical Trial	Progression-Free Survival (PFS)	Overall Survival (OS)
IMSPIRE-150 [29] (vemurafenib + cobimetinib)	Median (months) 10.6 5-year PFS NA	Not available
COMBI i [27] (dabrafenib + trametinib)	Median (months) 12 5-year PFS NA	Not available
COLOMBUS [28] (encorafenib + binimetinib)	Median (months) PFS 14.9 5-year PFS 23%	Median (months) 33.6 5-year OS 35%

**Table 2:** PFS and OS of immune checkpoint inhibitors (ICI).

Clinical Trial	Overall Response Rate (ORR)	Progression-Free Survival (PFS)	Overall Survival (OS)
CHECKMATE 67 Nivolumab (Anti-PD-1) [45]	45%	Median (months) 6.9 5-year PFS 29%	Median (months) 36.9 5-year OS 44%
CHECKMATE 67 Ipilimumab (anti-CTLA-4) [45]	19%	Median (months) 2.9 5-year PFS 7%	Median (months) 19.9 5-year OS 26%
CHECKMATE 67 Nivolumab + ipilimumab [45]	58%	Median (months) 11.5 5-year PFS 36%	Median (months) 72.1 5-year OS 52%

**Table 3:** PFS and OS of combined targeted and immunotherapy.

Clinical Trial	Overall Response Rate (ORR)	Progression-Free Survival (PFS)	Overall Survival (OS)
COMBI-I [51] Spartalizumab (anti-PD-1) + dabrafenib + trametinib	69%	Median (months) 16.2	Not available
IMSPIRE 150 [29] Atezolizumab (anti-PD-L1) + vemurafenib + cobimetinib	66%	Median (months) 15.1	Not available
KEYNOTE-022 [52] Pembrolizumab (anti-PD-1) + dabrafenib + trametinib	63%	Median (months) 16.9	Not available

## Data Availability

The data presented in this study are available on request from the corresponding author.

## References

[1] Ostrowski S.M., Fisher D.E. (2021). Biology of Melanoma. Hematol. Oncol. Clin. N. Am..

[2] Saginala K., Barsouk A., Aluru J.S., Rawla P., Barsouk A. (2021). Epidemiology of Melanoma. Med. Sci..

[3] Siegel R.L., Miller K.D., Jemal A. (2019). Cancer statistics, 2019. CA Cancer J. Clin..

[4] Domingues B., Lopes J., Soares P., Populo H. (2018). Melanoma treatment in review. Immunotargets Ther..

[5] Anderson C.M., Buzaid A.C., Legha S.S. (1995). Systemic treatments for advanced cutaneous melanoma. Oncology.

[6] Niault T.S., Baccarini M. (2010). Targets of Raf in tumorigenesis. Carcinogenesis.

[7] Bollag G., Tsai J., Zhang J., Zhang C., Ibrahim P., Nolop K., Hirth P. (2012). Vemurafenib: The first drug approved for BRAF-mutant cancer. Nat. Rev. Drug Discov..

[8] Dummer R., Ascierto P.A., Gogas H.J., Arance A., Mandala M., Liszkay G., Garbe C., Schadendorf D., Krajsova I., Gutzmer R. (2018). Overall survival in patients with BRAF-mutant melanoma receiving encorafenib plus binimetinib versus vemurafenib or encorafenib (COLUMBUS): A multicentre, open-label, randomised, phase 3 trial. Lancet Oncol..

[9] Larkin J., Ascierto P.A., Dréno B., Atkinson V., Liszkay G., Maio M., Mandalà M., Demidov L., Stroyakovskiy D., Thomas L. (2014). Combined Vemurafenib and Cobimetinib in *BRAF*-Mutated Melanoma. N. Engl. J. Med..

[10] Flaherty K.T., Robert C., Hersey P., Nathan P., Garbe C., Milhem M., Demidov L.V., Hassel J.C., Rutkowski P., Mohr P. (2012). Improved Survival with MEK Inhibition in BRAF-Mutated Melanoma. N. Engl. J. Med..

[11] Haanen J.B. (2013). Immunotherapy of melanoma. Eur. J. Cancer Suppl..

[12] Bollag G., Hirth P., Tsai J., Zhang J., Ibrahim P.N., Cho H., Spevak W., Zhang C., Zhang Y., Habets G. (2010). Clinical efficacy of a RAF inhibitor needs broad target blockade in BRAF-mutant melanoma. Nature.

[13] Søndergaard J.N., Nazarian R., Wang Q., Guo D., Hsueh T., Mok S., Sazegar H., MacConaill L.E., Barretina J.G., Kehoe S.M. (2010). Differential sensitivity of melanoma cell lines with BRAF V600E mutation to the specific Raf inhibitor PLX4032. J. Transl. Med..

[14] Yang H., Higgins B., Kolinsky K., Packman K., Go Z., Iyer R., Kolis S., Zhao S., Lee R., Grippo J.F. (2010). RG7204 (PLX4032), a Selective BRAFV600E Inhibitor, Displays Potent Antitumor Activity in Preclinical Melanoma Models. Cancer Res..

[15] Kloth J.S.L., Pagani A., Verboom M.C., Malovini A., Napolitano C., Kruit W.H., Sleijfer S., Steeghs N., Zambelli A., Mathijssen R.H.J. (2015). Incidence and relevance of QTc-interval prolongation caused by tyrosine kinase inhibitors. Br. J. Cancer.

[16] Chapman P.B., Hauschild A., Robert C., Haanen J.B., Ascierto P., Larkin J., Dummer R., Garbe C., Testori A., Maio M. (2011). Improved Survival with Vemurafenib in Melanoma with BRAF V600E Mutation. N. Engl. J. Med..

[17] Shirley M. (2018). Encorafenib and Binimetinib: First Global Approvals. Drugs.

[18] Sullivan R.J., Weber J., Patel S., Dummer R., Carlino M.S., Tan D.S.W., Lebbé C., Siena S., Elez E., Wollenberg L. (2020). A Phase Ib/II Study of the BRAF Inhibitor Encorafenib Plus the MEK Inhibitor Binimetinib in Patients with *BRAFV600E/K*-mutant Solid Tumors. Clin. Cancer Res..

[19] Long G.V., Stroyakovskiy D., Gogas H., Levchenko E., de Braud F., Larkin J., Garbe C., Jouary T., Hauschild A., Grob J.J. (2014). Combined BRAF and MEK Inhibition versus BRAF Inhibition Alone in Melanoma. N. Engl. J. Med..

[20] Long G.V., Stroyakovskiy D., Gogas H., Levchenko E., de Braud F., Larkin J., Garbe C., Jouary T., Hauschild A., Grob J.J. (2015). Dabrafenib and trametinib versus dabrafenib and placebo for Val600 BRAF-mutant melanoma: A multicentre, double-blind, phase 3 randomised controlled trial. Lancet.

[21] Nazarian R., Shi H., Wang Q., Kong X., Koya R.C., Lee H., Chen Z., Lee M.K., Attar N., Sazegar H. (2010). Melanomas acquire resistance to B-RAF(V600E) inhibition by RTK or N-RAS upregulation. Nature.

[22] Greger J.G., Eastman S.D., Zhang V., Bleam M.R., Hughes A.M., Smitheman K.N., Dickerson S.H., Laquerre S.G., Liu L., Gilmer T.M. (2012). Combinations of BRAF, MEK, and PI3K/mTOR Inhibitors Overcome Acquired Resistance to the BRAF Inhibitor GSK2118436 Dabrafenib, Mediated by *NRAS* or *MEK* Mutations. Mol. Cancer Ther..

[23] Wagle N., Emery C., Berger M.F., Davis M.J., Sawyer A., Pochanard P., Kehoe S.M., Johannessen C.M., Macconaill L.E., Hahn W.C. (2011). Dissecting Therapeutic Resistance to RAF Inhibition in Melanoma by Tumor Genomic Profiling. J. Clin. Oncol..

[24] Poulikakos P.I., Persaud Y., Janakiraman M., Kong X., Ng C., Moriceau G., Shi H., Atefi M., Titz B., Gabay M.T. (2011). RAF inhibitor resistance is mediated by dimerization of aberrantly spliced BRAF(V600E). Nature.

[25] Maertens O., Johnson B., Hollstein P., Frederick D.T., Cooper Z.A., Messiaen L., Bronson R.T., McMahon M., Granter S., Flaherty K. (2013). Elucidating Distinct Roles for *NF1* in Melanomagenesis. Cancer Discov..

[26] Wu P.-K., Park J.-I. (2015). MEK1/2 Inhibitors: Molecular Activity and Resistance Mechanisms. Semin. Oncol..

[27] Robert C., Grob J.J., Stroyakovskiy D., Karaszewska B., Hauschild A., Levchenko E., Chiarion Sileni V., Schachter J., Garbe C., Bondarenko I. (2019). Five-Year Outcomes with Dabrafenib plus Trametinib in Metastatic Melanoma. N. Engl. J. Med..

[28] Dummer R., Flaherty K.T., Robert C., Arance A., de Groot J.W.B., Garbe C., Gogas H.J., Gutzmer R., Krajsová I., Liszkay G. (2022). COLUMBUS 5-Year Update: A Randomized, Open-Label, Phase III Trial of Encorafenib Plus Binimetinib Versus Vemurafenib or Encorafenib in Patients with *BRAF* V600–Mutant Melanoma. J. Clin. Oncol..

[29] Gutzmer R., Stroyakovskiy D., Gogas H., Robert C., Lewis K., Protsenko S., Pereira R.P., Eigentler T., Rutkowski P., Demidov L. (2020). Atezolizumab, vemurafenib, and cobimetinib as first-line treatment for unresectable advanced BRAFV600 mutation-positive melanoma (IMspire150): Primary analysis of the randomised, double-blind, placebo-controlled, phase 3 trial. Lancet.

[30] Ott P.A., Hodi F.S., Robert C. (2013). CTLA-4 and PD-1/PD-L1 Blockade: New Immunotherapeutic Modalities with Durable Clinical Benefit in Melanoma Patients. Clin. Cancer Res..

[31] Walunas T.L., Lenschow D.J., Bakker C.Y., Linsley P.S., Freeman G.J., Green J.M., Thompson C.B., Bluestone J.A. (1994). CTLA-4 can function as a negative regulator of T cell activation. Immunity.

[32] Raskov H., Orhan A., Christensen J.P., Gögenur I. (2021). Cytotoxic CD8^+^ T cells in cancer and cancer immunotherapy. Br. J. Cancer.

[33] Korman A.J., Peggs K.S., Allison J.P. (2006). Checkpoint Blockade in Cancer Immunotherapy. Adv. Immunol..

[34] Schneider H., Downey J., Smith A., Zinselmeyer B.H., Rush C., Brewer J.M., Wei B., Hogg N., Garside P., Rudd C.E. (2006). Reversal of the TCR Stop Signal by CTLA-4. Science.

[35] Lanzavecchia A., Sallusto F. (2001). Antigen decoding by T lymphocytes: From synapses to fate determination. Nat. Immunol..

[36] Mellman I., Coukos G., Dranoff G. (2011). Cancer immunotherapy comes of age. Nature.

[37] Ishida Y., Agata Y., Shibahara K., Honjo T. (1992). Induced expression of PD-1, a novel member of the immunoglobulin gene superfamily, upon programmed cell death. EMBO J..

[38] Zou W., Chen L. (2008). Inhibitory B7-family molecules in the tumour microenvironment. Nat. Rev. Immunol..

[39] Arasanz H., Gato-Cañas M., Zuazo M., Ibañez-Vea M., Breckpot K., Kochan G., Escors D. (2017). PD1 signal transduction pathways in T cells. Oncotarget.

[40] Robert C., Karaszewska B., Schachter J., Rutkowski P., Mackiewicz A., Stroiakovski D., Lichinitser M., Dummer R., Grange F., Mortier L. (2015). Improved Overall Survival in Melanoma with Combined Dabrafenib and Trametinib. N. Engl. J. Med..

[41] Hamid O., Molinero L., Bolen C.R., Sosman J.A., Muñoz-Couselo E., Kluger H.M., McDermott D.F., Powderly J.D., Sarkar I., Ballinger M. (2019). Safety, Clinical Activity, and Biological Correlates of Response in Patients with Metastatic Melanoma: Results from a Phase I Trial of Atezolizumab. Clin. Cancer Res..

[42] Larkin J., Chiarion-Sileni V., Gonzalez R., Grob J.J., Rutkowski P., Lao C.D., Cowey C.L., Schadendorf D., Wagstaff J., Dummer R. (2019). Five-Year Survival with Combined Nivolumab and Ipilimumab in Advanced Melanoma. N. Engl. J. Med..

[43] Shahabi V., Whitney G., Hamid O., Schmidt H., Chasalow S.D., Alaparthy S., Jackson J.R. (2012). Assessment of association between BRAF-V600E mutation status in melanomas and clinical response to ipilimumab. Cancer Immunol. Immunother..

[44] Gide T.N., Wilmott J.S., Scolyer R.A., Long G.V. (2018). Primary and Acquired Resistance to Immune Checkpoint Inhibitors in Metastatic Melanoma. Clin. Cancer Res..

[45] Wolchok J.D., Chiarion-Sileni V., Gonzalez R., Grob J.J., Rutkowski P., Lao C.D., Cowey C.L., Schadendorf D., Wagstaff J., Dummer R. (2022). Long-Term Outcomes with Nivolumab Plus Ipilimumab or Nivolumab Alone Versus Ipilimumab in Patients with Advanced Melanoma. J. Clin. Oncol..

[46] Pelster M.S., Amaria R.N. (2019). Combined targeted therapy and immunotherapy in melanoma: A review of the impact on the tumor microenvironment and outcomes of early clinical trials. Ther. Adv. Med. Oncol..

[47] Kersh A.E., Ng S., Chang Y.M., Sasaki M., Thomas S.N., Kissick H.T., Lesinski G.B., Kudchadkar R.R., Waller E.K., Pollack B.P. (2018). Targeted Therapies: Immunologic Effects and Potential Applications Outside of Cancer. J. Clin. Pharmacol..

[48] Ribas A., Hodi F.S., Callahan M., Konto C., Wolchok J. (2013). Hepatotoxicity with Combination of Vemurafenib and Ipilimumab. N. Engl. J. Med..

[49] Minor D.R., Puzanov I., Callahan M.K., Hug B.A., Hoos A. (2015). Severe gastrointestinal toxicity with administration of trametinib in combination with dabrafenib and ipilimumab. Pigment Cell Melanoma Res..

[50] Huynh S., Mortier L., Dutriaux C., Maubec E., Boileau M., Dereure O., Leccia M.T., Arnault J.P., Brunet-Possenti F., Aubin F. (2020). Combined Therapy with Anti-PD1 and BRAF and/or MEK Inhibitor for Advanced Melanoma: A Multicenter Cohort Study. Cancers.

[51] Dummer R., Lebbé C., Atkinson V., Mandalà M., Nathan P.D., Arance A., Richtig E., Yamazaki N., Robert C., Schadendorf D. (2020). Combined PD-1, BRAF and MEK inhibition in advanced BRAF-mutant melanoma: Safety run-in and biomarker cohorts of COMBI-i. Nat. Med..

[52] Ferrucci P.F., Di Giacomo A.M., Del Vecchio M., Atkinson V., Schmidt H., Schachter J., Queirolo P., Long G.V., Stephens R., Svane I.M. (2020). KEYNOTE-022 part 3: A randomized, double-blind, phase 2 study of pembrolizumab, dabrafenib, and trametinib in *BRAF*-mutant melanoma. J. Immunother. Cancer.

[53] Pavlick A.C., Fecher L., Ascierto P.A., Sullivan R.J. (2019). Frontline Therapy for *BRAF*-Mutated Metastatic Melanoma: How Do You Choose, and Is There One Correct Answer?. Am. Soc. Clin. Oncol. Educ. Book.

[54] Johnson D.B., Pectasides E., Feld E., Ye F., Zhao S., Johnpulle R., Merritt R., McDermott D.F., Puzanov I., Lawrence D. (2017). Sequencing Treatment in BRAF V600 Mutant Melanoma: Anti-PD-1 Before and After BRAF Inhibition. J. Immunother..

[55] Saab K.R., Mooradian M.J., Wang D.Y., Chon J., Xia C.Y., Bialczak A., Abbate K.T., Menzies A.M., Johnson D.B., Sullivan R.J. (2019). Tolerance and efficacy of BRAF plus MEK inhibition in patients with melanoma who previously have received programmed cell death protein 1-based therapy. Cancer.

[56] Hugo W., Zaretsky J.M., Sun L., Song C., Moreno B.H., Hu-Lieskovan S., Berent-Maoz B., Pang J., Chmielowski B., Cherry G. (2016). Genomic and Transcriptomic Features of Response to Anti-PD-1 Therapy in Metastatic Melanoma. Cell.

[57] Atkins M.B., Lee S.J., Chmielowski B., Tarhini A.A., Cohen G.I., Truong T.G., Moon H.H., Davar D., O’Rourke M., Stephenson J.J. (2023). Combination Dabrafenib and Trametinib Versus Combination Nivolumab and Ipilimumab for Patients with Advanced *BRAF* -Mutant Melanoma: The DREAMseq Trial—ECOG-ACRIN EA6134. J. Clin. Oncol..

[58] Ascierto P.A., Bastholt L., Ferrucci P.F., Hansson J., Márquez Rodas I., Payne M., Robert C., Thomas L., Utikal J.S., Wolter P. (2018). The impact of patient characteristics and disease-specific factors on first-line treatment decisions for BRAF-mutated melanoma: Results from a European expert panel study. Melanoma Res..

[59] Buder-Bakhaya K., Hassel J.C. (2018). Biomarkers for Clinical Benefit of Immune Checkpoint Inhibitor Treatment—A Review from the Melanoma Perspective and Beyond. Front. Immunol..

[60] Fendt S.-M., Frezza C., Erez A. (2020). Targeting Metabolic Plasticity and Flexibility Dynamics for Cancer Therapy. Cancer Discov..

[61] Hanahan D., Weinberg R.A. (2011). Hallmarks of Cancer: The Next Generation. Cell.

[62] Fischer G.M., Vashisht Gopal Y.N., McQuade J.L., Peng W., DeBerardinis R.J., Davies M.A. (2018). Metabolic strategies of melanoma cells: Mechanisms, interactions with the tumor microenvironment, and therapeutic implications. Pigment Cell Melanoma Res..

[63] Baudy A.R., Dogan T., Flores-Mercado J.E., Hoeflich K.P., Su F., van Bruggen N., Williams S.P. (2012). FDG-PET is a good biomarker of both early response and acquired resistance in BRAFV600 mutant melanomas treated with vemurafenib and the MEK inhibitor GDC-0973. EJNMMI Res..

[64] McArthur G.A., Puzanov I., Amaravadi R., Ribas A., Chapman P., Kim K.B., Sosman J.A., Lee R.J., Nolop K., Flaherty K.T. (2012). Marked, Homogeneous, and Early [^18^F]Fluorodeoxyglucose–Positron Emission Tomography Responses to Vemurafenib in *BRAF* -Mutant Advanced Melanoma. J. Clin. Oncol..

[65] Khamari R., Trinh A., Gabert P.E., Corazao-Rozas P., Riveros-Cruz S., Balayssac S., Malet-Martino M., Dekiouk S., Joncquel Chevalier Curt M., Maboudou P. (2018). Glucose metabolism and NRF2 coordinate the antioxidant response in melanoma resistant to MAPK inhibitors. Cell Death Dis..

[66] Baenke F., Chaneton B., Smith M., Van Den Broek N., Hogan K., Tang H., Viros A., Martin M., Galbraith L., Girotti M.R. (2016). Resistance to BRAF inhibitors induces glutamine dependency in melanoma cells. Mol. Oncol..

[67] Hernandez-Davies J.E., Tran T.Q., Reid M.A., Rosales K.R., Lowman X.H., Pan M., Moriceau G., Yang Y., Wu J., Lo R.S. (2015). Vemurafenib resistance reprograms melanoma cells towards glutamine dependence. J. Transl. Med..

[68] Singleton K.R., Crawford L., Tsui E., Manchester H.E., Maertens O., Liu X., Liberti M.V., Magpusao A.N., Stein E.M., Tingley J.P. (2017). Melanoma Therapeutic Strategies that Select against Resistance by Exploiting MYC-Driven Evolutionary Convergence. Cell Rep..

[69] Ross K.C., Andrews A.J., Marion C.D., Yen T.J., Bhattacharjee V. (2017). Identification of the Serine Biosynthesis Pathway as a Critical Component of BRAF Inhibitor Resistance of Melanoma, Pancreatic, and Non–Small Cell Lung Cancer Cells. Mol. Cancer Ther..

[70] Corazao-Rozas P., Guerreschi P., Jendoubi M., André F., Jonneaux A., Scalbert C., Garçon G., Malet-Martino M., Balayssac S., Rocchi S. (2013). Mitochondrial oxidative stress is the achille’s heel of melanoma cells resistant to Braf-mutant inhibitor. Oncotarget.

[71] Haq R., Fisher D.E., Widlund H.R. (2014). Molecular Pathways: BRAF Induces Bioenergetic Adaptation by Attenuating Oxidative Phosphorylation. Clin. Cancer Res..

[72] Warrier G., Lanceta L., Imbert-Fernandez Y., Chesney J.A. (2019). Inhibition of glucose metabolism through treatment of BRAF mutated metastatic melanoma with vemurafenib. J. Clin. Oncol..

[73] Hardeman K.N., Peng C., Paudel B.B., Meyer C.T., Luong T., Tyson D.R., Young J.D., Quaranta V., Fessel J.P. (2017). Dependence on Glycolysis Sensitizes BRAF-mutated Melanomas for Increased Response to Targeted BRAF Inhibition. Sci. Rep..

[74] Delgado-Goni T., Miniotis M.F., Wantuch S., Parkes H.G., Marais R., Workman P., Leach M.O., Beloueche-Babari M. (2016). The BRAF Inhibitor Vemurafenib Activates Mitochondrial Metabolism and Inhibits Hyperpolarized Pyruvate–Lactate Exchange in BRAF-Mutant Human Melanoma Cells. Mol. Cancer Ther..

[75] Parmenter T.J., Kleinschmidt M., Kinross K.M., Bond S.T., Li J., Kaadige M.R., Rao A., Sheppard K.E., Hugo W., Pupo G.M. (2014). Response of *BRAF*-Mutant Melanoma to BRAF Inhibition Is Mediated by a Network of Transcriptional Regulators of Glycolysis. Cancer Discov..

[76] Hosios A.M., Hecht V.C., Danai L.V., Johnson M.O., Rathmell J.C., Steinhauser M.L., Manalis S.R., Vander Heiden M.G. (2016). Amino Acids Rather than Glucose Account for the Majority of Cell Mass in Proliferating Mammalian Cells. Dev. Cell.

[77] Eagle H. (1955). Nutrition Needs of Mammalian Cells in Tissue Culture. Science.

[78] Cormerais Y., Massard P.A., Vucetic M., Giuliano S., Tambutté E., Durivault J., Vial V., Endou H., Wempe M.F., Parks S.K. (2018). The glutamine transporter ASCT2 (SLC1A5) promotes tumor growth independently of the amino acid transporter LAT1 (SLC7A5). J. Biol. Chem..

[79] Scott D.A., Richardson A.D., Filipp F.V., Knutzen C.A., Chiang G.G., Ronai Z.A., Osterman A.L., Smith J.W. (2011). Comparative Metabolic Flux Profiling of Melanoma Cell Lines. J. Biol. Chem..

[80] Gao P., Tchernyshyov I., Chang T.C., Lee Y.S., Kita K., Ochi T., Zeller K.I., De Marzo A.M., Van Eyk J.E., Mendell J.T. (2009). c-Myc suppression of miR-23a/b enhances mitochondrial glutaminase expression and glutamine metabolism. Nature.

[81] Wise D.R., DeBerardinis R.J., Mancuso A., Sayed N., Zhang X.Y., Pfeiffer H.K., Nissim I., Daikhin E., Yudkoff M., McMahon S.B. (2008). Myc regulates a transcriptional program that stimulates mitochondrial glutaminolysis and leads to glutamine addiction. Proc. Natl. Acad. Sci. USA.

[82] Possemato R., Marks K.M., Shaul Y.D., Pacold M.E., Kim D., Birsoy K., Sethumadhavan S., Woo H.K., Jang H.G., Jha A.K. (2011). Functional genomics reveal that the serine synthesis pathway is essential in breast cancer. Nature.

[83] Ravez S., Spillier Q., Marteau R., Feron O., Frédérick R. (2017). Challenges and Opportunities in the Development of Serine Synthetic Pathway Inhibitors for Cancer Therapy. J. Med. Chem..

[84] Mullarky E., Mattaini K.R., Vander Heiden M.G., Cantley L.C., Locasale J.W. (2011). *PHGDH* amplification and altered glucose metabolism in human melanoma. Pigment Cell Melanoma Res..

[85] Garcia-Bermudez J., Baudrier L., La K., Zhu X.G., Fidelin J., Sviderskiy V.O., Papagiannakopoulos T., Molina H., Snuderl M., Lewis C.A. (2018). Aspartate is a limiting metabolite for cancer cell proliferation under hypoxia and in tumours. Nat. Cell Biol..

[86] Jain M., Nilsson R., Sharma S., Madhusudhan N., Kitami T., Souza A.L., Kafri R., Kirschner M.W., Clish C.B., Mootha V.K. (2012). Metabolite Profiling Identifies a Key Role for Glycine in Rapid Cancer Cell Proliferation. Science.

[87] Sonveaux P., Végran F., Schroeder T., Wergin M.C., Verrax J., Rabbani Z.N., De Saedeleer C.J., Kennedy K.M., Diepart C., Jordan B.F. (2008). Targeting lactate-fueled respiration selectively kills hypoxic tumor cells in mice. J. Clin. Investig..

[88] Feron O. (2009). Pyruvate into lactate and back: From the Warburg effect to symbiotic energy fuel exchange in cancer cells. Radiother. Oncol..

[89] Sahai E., Astsaturov I., Cukierman E., DeNardo D.G., Egeblad M., Evans R.M., Fearon D., Greten F.R., Hingorani S.R., Hunter T. (2020). A framework for advancing our understanding of cancer-associated fibroblasts. Nat. Rev. Cancer.

[90] Kumar P.R., Moore J.A., Bowles K.M., Rushworth S.A., Moncrieff M.D. (2021). Mitochondrial oxidative phosphorylation in cutaneous melanoma. Br. J. Cancer.

[91] Haq R., Shoag J., Andreu-Perez P., Yokoyama S., Edelman H., Rowe G.C., Frederick D.T., Hurley A.D., Nellore A., Kung A.L. (2013). Oncogenic BRAF Regulates Oxidative Metabolism via PGC1α and MITF. Cancer Cell.

[92] Kumar S.M., Yu H., Edwards R., Chen L., Kazianis S., Brafford P., Acs G., Herlyn M., Xu X. (2007). Mutant V600E *BRAF* Increases Hypoxia Inducible Factor-1α Expression in Melanoma. Cancer Res..

[93] Hoffman B., Liebermann D.A. (2008). Apoptotic signaling by c-MYC. Oncogene.

[94] Lin X., Sun R., Zhao X., Zhu D., Zhao X., Gu Q., Dong X., Zhang D., Zhang Y., Li Y. (2017). C-myc overexpression drives melanoma metastasis by promoting vasculogenic mimicry via c-myc/snail/Bax signaling. J. Mol. Med..

[95] Sears R., Nuckolls F., Haura E., Taya Y., Tamai K., Nevins J.R. (2000). Multiple Ras-dependent phosphorylation pathways regulate Myc protein stability. Genes Dev..

[96] Osthus R.C., Shim H., Kim S., Li Q., Reddy R., Mukherjee M., Xu Y., Wonsey D., Lee L.A., Dang C.V. (2000). Deregulation of Glucose Transporter 1 and Glycolytic Gene Expression by c-Myc. J. Biol. Chem..

[97] Shim H., Dolde C., Lewis B.C., Wu C.S., Dang G., Jungmann R.A., Dalla-Favera R., Dang C.V. (1997). c-Myc transactivation of *LDH-A*: Implications for tumor metabolism and growth. Proc. Natl. Acad. Sci. USA.

[98] Doherty J.R., Yang C., Scott K.E., Cameron M.D., Fallahi M., Li W., Hall M.A., Amelio A.L., Mishra J.K., Li F. (2014). Blocking Lactate Export by Inhibiting the Myc Target MCT1 Disables Glycolysis and Glutathione Synthesis. Cancer Res..

[99] Marin-Hernandez A., Gallardo-Perez J., Ralph S., Rodriguez-Enriquez S., Moreno-Sanchez R. (2009). HIF-1α Modulates Energy Metabolism in Cancer Cells by Inducing Over-Expression of Specific Glycolytic Isoforms. Mini-Rev. Med. Chem..

[100] Semenza G.L., Roth P.H., Fang H.M., Wang G.L. (1994). Transcriptional regulation of genes encoding glycolytic enzymes by hypoxia-inducible factor 1. J. Biol. Chem..

[101] Kim J., Tchernyshyov I., Semenza G.L., Dang C.V. (2006). HIF-1-mediated expression of pyruvate dehydrogenase kinase: A metabolic switch required for cellular adaptation to hypoxia. Cell Metab..

[102] Kuphal S., Winklmeier A., Warnecke C., Bosserhoff A.-K. (2010). Constitutive HIF-1 activity in malignant melanoma. Eur. J. Cancer.

[103] Mahmood N., Mihalcioiu C., Rabbani S.A. (2018). Multifaceted Role of the Urokinase-Type Plasminogen Activator (uPA) and Its Receptor (uPAR): Diagnostic, Prognostic, and Therapeutic Applications. Front. Oncol..

[104] Rofstad E.K., Mathiesen B., Galappathi K. (2004). Increased Metastatic Dissemination in Human Melanoma Xenografts after Subcurative Radiation Treatment. Cancer Res..

[105] Laurenzana A., Chillà A., Luciani C., Peppicelli S., Biagioni A., Bianchini F., Tenedini E., Torre E., Mocali A., Calorini L. (2017). uPA/uPAR system activation drives a glycolytic phenotype in melanoma cells. Int. J. Cancer.

[106] Pinheiro C., Miranda-Gonçalves V., Longatto-Filho A., Vicente A.L., Berardinelli G.N., Scapulatempo-Neto C., Costa R.F., Viana C.R., Reis R.M., Baltazar F. (2016). The metabolic microenvironment of melanomas: Prognostic value of MCT1 and MCT4. Cell Cycle.

[107] Jin J., Byun J.-K., Choi Y.-K., Park K.-G. (2023). Targeting glutamine metabolism as a therapeutic strategy for cancer. Exp. Mol. Med..

[108] Varghese S., Pramanik S., Williams L.J., Hodges H.R., Hudgens C.W., Fischer G.M., Luo C.K., Knighton B., Tan L., Lorenzi P.L. (2021). The Glutaminase Inhibitor CB-839 (Telaglenastat) Enhances the Antimelanoma Activity of T-Cell–Mediated Immunotherapies. Mol. Cancer Ther..

[109] Krakowski I., Häbel H., Nielsen K., Ingvar C., Andersson T.M.L., Girnita A., Smedby K.E., Eriksson H. (2023). Association of metformin use and survival in patients with cutaneous melanoma and diabetes. Br. J. Dermatol..

[110] Yuan P., Ito K., Perez-Lorenzo R., Del Guzzo C., Lee J.H., Shen C.H., Bosenberg M.W., McMahon M., Cantley L.C., Zheng B. (2013). Phenformin enhances the therapeutic benefit of BRAF ^V600E^ inhibition in melanoma. Proc. Natl. Acad. Sci. USA.

[111] Pollak M.N. (2012). Investigating Metformin for Cancer Prevention and Treatment: The End of the Beginning. Cancer Discov..

[112] Zheng B., Jeong J.H., Asara J.M., Yuan Y.Y., Granter S.R., Chin L., Cantley L.C. (2009). Oncogenic B-RAF Negatively Regulates the Tumor Suppressor LKB1 to Promote Melanoma Cell Proliferation. Mol. Cell.

[113] Montaudié H., Cerezo M., Bahadoran P., Roger C., Passeron T., Machet L., Arnault J.P., Verneuil L., Maubec E., Aubin F. (2017). Metformin monotherapy in melanoma: A pilot, open-label, prospective, and multicentric study indicates no benefit. Pigment Cell Melanoma Res..

[114] Martin M.J., Hayward R., Viros A., Marais R. (2012). Metformin Accelerates the Growth of BRAFV600E-Driven Melanoma by Upregulating VEGF-A. Cancer Discov..

[115] Vandyck H.H., Hillen L.M., Bosisio F.M., van den Oord J., zur Hausen A., Winnepenninckx V. (2021). Rethinking the biology of metastatic melanoma: A holistic approach. Cancer Metastasis Rev..

[116] Ghesquière B., Wong B.W., Kuchnio A., Carmeliet P. (2014). Metabolism of stromal and immune cells in health and disease. Nature.

[117] Rangel Rivera G.O., Knochelmann H.M., Dwyer C.J., Smith A.S., Wyatt M.M., Rivera-Reyes A.M., Thaxton J.E., Paulos C.M. (2021). Fundamentals of T Cell Metabolism and Strategies to Enhance Cancer Immunotherapy. Front. Immunol..

[118] Pearce E.L., Poffenberger M.C., Chang C.-H., Jones R.G. (2013). Fueling Immunity: Insights into Metabolism and Lymphocyte Function. Science.

[119] Xia L., Oyang L., Lin J., Tan S., Han Y., Wu N., Yi P., Tang L., Pan Q., Rao S. (2021). The cancer metabolic reprogramming and immune response. Mol. Cancer.

[120] Chang C.-H., Qiu J., O’Sullivan D., Buck M.D., Noguchi T., Curtis J.D., Chen Q., Gindin M., Gubin M.M., van der Windt G.J. (2015). Metabolic Competition in the Tumor Microenvironment Is a Driver of Cancer Progression. Cell.

[121] Na K.J., Choi H., Oh H.R., Kim Y.H., Lee S.B., Jung Y.J., Koh J., Park S., Lee H.J., Jeon Y.K. (2020). Reciprocal change in Glucose metabolism of Cancer and Immune Cells mediated by different Glucose Transporters predicts Immunotherapy response. Theranostics.

[122] Jacobs S.R., Herman C.E., Maciver N.J., Wofford J.A., Wieman H.L., Hammen J.J., Rathmell J.C. (2008). Glucose Uptake Is Limiting in T Cell Activation and Requires CD28-Mediated Akt-Dependent and Independent Pathways. J. Immunol..

[123] Frauwirth K.A., Riley J.L., Harris M.H., Parry R.V., Rathmell J.C., Plas D.R., Elstrom R.L., June C.H., Thompson C.B. (2002). The CD28 Signaling Pathway Regulates Glucose Metabolism. Immunity.

[124] Cham C.M., Driessens G., O’Keefe J.P., Gajewski T.F. (2008). Glucose deprivation inhibits multiple key gene expression events and effector functions in CD8^+^ T cells. Eur. J. Immunol..

[125] Najjar Y.G., Menk A.V., Sander C., Rao U., Karunamurthy A., Bhatia R., Zhai S., Kirkwood J.M., Delgoffe G.M. (2019). Tumor cell oxidative metabolism as a barrier to PD-1 blockade immunotherapy in melanoma. JCI Insight.

[126] Singer K., Kastenberger M., Gottfried E., Hammerschmied C.G., Büttner M., Aigner M., Seliger B., Walter B., Schlösser H., Hartmann A. (2011). Warburg phenotype in renal cell carcinoma: High expression of glucose-transporter 1 (GLUT-1) correlates with low CD8^+^ T-cell infiltration in the tumor. Int. J. Cancer.

[127] Sukumar M., Roychoudhuri R., Restifo N.P. (2015). Nutrient Competition: A New Axis of Tumor Immunosuppression. Cell.

[128] Gottfried E., Kunz-Schughart L.A., Ebner S., Mueller-Klieser W., Hoves S., Andreesen R., Mackensen A., Kreutz M. (2006). Tumor-derived lactic acid modulates dendritic cell activation and antigen expression. Blood.

[129] Harmon C., O’Farrelly C., Robinson M.W. (2020). The Immune Consequences of Lactate in the Tumor Microenvironment. Tumor Microenvironment.

[130] Fischer K., Hoffmann P., Voelkl S., Meidenbauer N., Ammer J., Edinger M., Gottfried E., Schwarz S., Rothe G., Hoves S. (2007). Inhibitory effect of tumor cell–derived lactic acid on human T cells. Blood.

[131] Ohta A., Madasu M., Subramanian M., Kini R., Jones G., Choukèr A., Ohta A., Sitkovsky M. (2014). Hypoxia-induced and A2A adenosine receptor-independent T-cell suppression is short lived and easily reversible. Int. Immunol..

[132] Ma X., Bi E., Lu Y., Su P., Huang C., Liu L., Wang Q., Yang M., Kalady M.F., Qian J. (2019). Cholesterol Induces CD8^+^ T Cell Exhaustion in the Tumor Microenvironment. Cell Metab..

[133] Yu Y., Liang Y., Li D., Wang L., Liang Z., Chen Y., Ma G., Wu H., Jiao W., Niu H. (2021). Glucose metabolism involved in PD-L1-mediated immune escape in the malignant kidney tumour microenvironment. Cell Death Discov..

[134] Atefi M., Avramis E., Lassen A., Wong D.J., Robert L., Foulad D., Cerniglia M., Titz B., Chodon T., Graeber T.G. (2014). Effects of MAPK and PI3K Pathways on PD-L1 Expression in Melanoma. Clin. Cancer Res..

[135] Onishi H., Fujimura A., Oyama Y., Yamasaki A., Imaizumi A., Kawamoto M., Katano M., Umebayashi M., Morisaki T. (2016). Hedgehog signaling regulates PDL-1 expression in cancer cells to induce anti-tumor activity by activated lymphocytes. Cell Immunol..

[136] Ingham P.W., McMahon A.P. (2001). Hedgehog signaling in animal development: Paradigms and principles. Genes Dev..

[137] Patsoukis N., Bardhan K., Chatterjee P., Sari D., Liu B., Bell L.N., Karoly E.D., Freeman G.J., Petkova V., Seth P. (2015). PD-1 alters T-cell metabolic reprogramming by inhibiting glycolysis and promoting lipolysis and fatty acid oxidation. Nat. Commun..

[138] Basu S., Hess S., Nielsen Braad P.-E., Olsen B.B., Inglev S., Høilund-Carlsen P.F. (2014). The Basic Principles of FDG-PET/CT Imaging. PET Clin..

[139] Carlino M.S., Saunders C.A., Haydu L.E., Menzies A.M., Martin Curtis C., Lebowitz P.F., Kefford R.F., Long G.V. (2013). ^18^F-labelled fluorodeoxyglucose–positron emission tomography (FDG–PET) heterogeneity of response is prognostic in dabrafenib treated BRAF mutant metastatic melanoma. Eur. J. Cancer.

[140] Sachpekidis C., Larribere L., Pan L., Haberkorn U., Dimitrakopoulou-Strauss A., Hassel J.C. (2015). Predictive value of early ^18^F-FDG PET/CT studies for treatment response evaluation to ipilimumab in metastatic melanoma: Preliminary results of an ongoing study. Eur. J. Nucl. Med. Mol. Imaging.

[141] Mangas Losada M., Romero Robles L., Mendoza Melero A., García Megías I., Villanueva Torres A., Garrastachu Zumarán P., Boulvard Chollet X., Lopci E., Ramírez Lasanta R., Delgado Bolton R.C. (2023). [^18^F]FDG PET/CT in the Evaluation of Melanoma Patients Treated with Immunotherapy. Diagnostics.

[142] Bier G., Hoffmann V., Kloth C., Othman A.E., Eigentler T., Garbe C., La Fougère C., Pfannenberg C., Nikolaou K., Klumpp B. (2016). CT imaging of bone and bone marrow infiltration in malignant melanoma—Challenges and limitations for clinical staging in comparison to 18FDG-PET/CT. Eur. J. Radiol..

[143] Kong B.Y., Menzies A.M., Saunders C.A., Liniker E., Ramanujam S., Guminski A., Kefford R.F., Long G.V., Carlino M.S. (2016). Residual FDG-PET metabolic activity in metastatic melanoma patients with prolonged response to anti-PD-1 therapy. Pigment Cell Melanoma Res..

[144] Anwar H., Sachpekidis C., Winkler J., Kopp-Schneider A., Haberkorn U., Hassel J.C., Dimitrakopoulou-Strauss A. (2018). Absolute number of new lesions on 18F-FDG PET/CT is more predictive of clinical response than SUV changes in metastatic melanoma patients receiving ipilimumab. Eur. J. Nucl. Med. Mol. Imaging.

[145] Berz A.M., Dromain C., Vietti-Violi N., Boughdad S., Duran R. (2022). Tumor response assessment on imaging following immunotherapy. Front. Oncol..

[146] Goldfarb L., Duchemann B., Chouahnia K., Zelek L., Soussan M. (2019). Monitoring anti-PD-1-based immunotherapy in non-small cell lung cancer with FDG PET: Introduction of iPERCIST. EJNMMI Res..

[147] Ito K., Teng R., Schöder H., Humm J.L., Ni A., Michaud L., Nakajima R., Yamashita R., Wolchok J.D., Weber W.A. (2019). ^18^F-FDG PET/CT for Monitoring of Ipilimumab Therapy in Patients with Metastatic Melanoma. J. Nucl. Med..

[148] Filippi L., Bianconi F., Schillaci O., Spanu A., Palumbo B. (2022). The Role and Potential of ^18^F-FDG PET/CT in Malignant Melanoma: Prognostication, Monitoring Response to Targeted and Immunotherapy, and Radiomics. Diagnostics.

[149] Faubert B., Tasdogan A., Morrison S.J., Mathews T.P., DeBerardinis R.J. (2021). Stable isotope tracing to assess tumor metabolism in vivo. Nat. Protoc..

[150] Giraudeau P. (2020). NMR-based metabolomics and fluxomics: Developments and future prospects. Analyst.

[151] Bartman C.R., Faubert B., Rabinowitz J.D., DeBerardinis R.J. (2023). Metabolic pathway analysis using stable isotopes in patients with cancer. Nat. Rev. Cancer.

[152] Fan T.W., Lane A.N., Higashi R.M., Farag M.A., Gao H., Bousamra M., Miller D.M. (2009). Altered regulation of metabolic pathways in human lung cancer discerned by ^13^C stable isotope-resolved metabolomics (SIRM). Mol. Cancer.

[153] Maher E.A., Marin-Valencia I., Bachoo R.M., Mashimo T., Raisanen J., Hatanpaa K.J., Jindal A., Jeffrey F.M., Choi C., Madden C. (2012). Metabolism of [U-^13^C]glucose in human brain tumors in vivo. NMR Biomed..

[154] Antoniewicz M.R. (2018). A guide to ^13^C metabolic flux analysis for the cancer biologist. Exp. Mol. Med..

[155] Wang Z.J., Ohliger M.A., Larson P.E.Z., Gordon J.W., Bok R.A., Slater J., Villanueva-Meyer J.E., Hess C.P., Kurhanewicz J., Vigneron D.B. (2019). Hyperpolarized ^13^C MRI: State of the Art and Future Directions. Radiology.

[156] Deen S.S., Rooney C., Shinozaki A., McGing J., Grist J.T., Tyler D.J., Serrão E., Gallagher F.A. (2023). Hyperpolarized Carbon 13 MRI: Clinical Applications and Future Directions in Oncology. Radiol. Imaging Cancer.

[157] Dutta P., Salzillo T.C., Pudakalakatti S., Gammon S.T., Kaipparettu B.A., McAllister F., Wagner S., Frigo D.E., Logothetis C.J., Zacharias N.M. (2019). Assessing Therapeutic Efficacy in Real-time by Hyperpolarized Magnetic Resonance Metabolic Imaging. Cells.

[158] Zaccagna F., Grist J.T., Deen S.S., Woitek R., Lechermann L.M., McLean M.A., Basu B., Gallagher F.A. (2018). Hyperpolarized carbon-13 magnetic resonance spectroscopic imaging: A clinical tool for studying tumour metabolism. Br. J. Radiol..

[159] Ursprung S., Woitek R., McLean M.A., Priest A.N., Crispin-Ortuzar M., Brodie C.R., Gill A.B., Gehrung M., Beer L., Riddick A.C.P. (2022). Hyperpolarized ^13^C-Pyruvate Metabolism as a Surrogate for Tumor Grade and Poor Outcome in Renal Cell Carcinoma—A Proof of Principle Study. Cancers.

[160] Jørgensen S.H., Bøgh N., Hansen E.S.S., Væggemose M., Wiggers H., Laustsen C. (2022). Hyperpolarized MRI—An Update and Future Perspectives. Semin. Nucl. Med..

[161] Carneiro T.J., Pinto J., Serrao E.M., Barros A.S., Brindle K.M., Gil A.M. (2022). Metabolic profiling of induced acute pancreatitis and pancreatic cancer progression in a mutant Kras mouse model. Front. Mol. Biosci..

[162] Rao Y., Gammon S., Zacharias N.M., Liu T., Salzillo T., Xi Y., Wang J., Bhattacharya P., Piwnica-Worms D. (2020). Hyperpolarized [1-^13^C]pyruvate-to-[1-^13^C]lactate conversion is rate-limited by monocarboxylate transporter-1 in the plasma membrane. Proc. Natl. Acad. Sci. USA.

[163] Woitek R., McLean M.A., Gill A.B., Grist J.T., Provenzano E., Patterson A.J., Ursprung S., Torheim T., Zaccagna F., Locke M. (2020). Hyperpolarized ^13^C MRI of Tumor Metabolism Demonstrates Early Metabolic Response to Neoadjuvant Chemotherapy in Breast Cancer. Radiol. Imaging Cancer.

[164] Nelson S.J., Kurhanewicz J., Vigneron D.B., Larson P.E., Harzstark A.L., Ferrone M., van Criekinge M., Chang J.W., Bok R., Park I. (2013). Metabolic Imaging of Patients with Prostate Cancer Using Hyperpolarized [1-^13^C]Pyruvate. Sci. Transl. Med..

[165] Miloushev V.Z., Granlund K.L., Boltyanskiy R., Lyashchenko S.K., DeAngelis L.M., Mellinghoff I.K., Brennan C.W., Tabar V., Yang T.J., Holodny A.I. (2018). Metabolic Imaging of the Human Brain with Hyperpolarized ^13^C Pyruvate Demonstrates ^13^C Lactate Production in Brain Tumor Patients. Cancer Res..

[166] Zaccagna F., McLean M.A., Grist J.T., Kaggie J., Mair R., Riemer F., Woitek R., Gill A.B., Deen S., Daniels C.J. (2022). Imaging Glioblastoma Metabolism by Using Hyperpolarized [1-^13^C]Pyruvate Demonstrates Heterogeneity in Lactate Labeling: A Proof of Principle Study. Radiol. Imaging Cancer.

[167] Albers M.J., Bok R., Chen A.P., Cunningham C.H., Zierhut M.L., Zhang V.Y., Kohler S.J., Tropp J., Hurd R.E., Yen Y.F. (2008). Hyperpolarized ^13^C Lactate, Pyruvate, and Alanine: Noninvasive Biomarkers for Prostate Cancer Detection and Grading. Cancer Res..

[168] Granlund K.L., Tee S.S., Vargas H.A., Lyashchenko S.K., Reznik E., Fine S., Laudone V., Eastham J.A., Touijer K.A., Reuter V.E. (2020). Hyperpolarized MRI of Human Prostate Cancer Reveals Increased Lactate with Tumor Grade Driven by Monocarboxylate Transporter 1. Cell Metab..

[169] Aggarwal R., Vigneron D.B., Kurhanewicz J. (2017). Hyperpolarized 1-[^13^C]-Pyruvate Magnetic Resonance Imaging Detects an Early Metabolic Response to Androgen Ablation Therapy in Prostate Cancer. Eur. Urol..

[170] Autry A.W., Gordon J.W., Chen H.Y., LaFontaine M., Bok R., Van Criekinge M., Slater J.B., Carvajal L., Villanueva-Meyer J.E., Chang S.M. (2020). Characterization of serial hyperpolarized ^13^C metabolic imaging in patients with glioma. Neuroimage Clin..

[171] Chen H.-Y., Aggarwal R., Bok R.A., Ohliger M.A., Zhu Z., Lee P., Gordon J.W., van Criekinge M., Carvajal L., Slater J.B. (2020). Hyperpolarized ^13^C-pyruvate MRI detects real-time metabolic flux in prostate cancer metastases to bone and liver: A clinical feasibility study. Prostate Cancer Prostatic Dis..

[172] Acciardo S., Mignion L., Lacomblez E., Schoonjans C., Joudiou N., Gourgue F., Bouzin C., Baurain J.F., Gallez B., Jordan B.F. (2020). Metabolic imaging using hyperpolarized ^13^C-pyruvate to assess sensitivity to the B-Raf inhibitor vemurafenib in melanoma cells and xenografts. J. Cell Mol. Med..

[173] Meeth K., Wang J.X., Micevic G., Damsky W., Bosenberg M.W. (2016). The YUMMlines: A series of congenic mouse melanoma cell lines with defined genetic alterations. Pigment Cell Melanoma Res..

[174] Wang J., Perry C.J., Meeth K., Thakral D., Damsky W., Micevic G., Kaech S., Blenman K., Bosenberg M. (2017). UV-induced somatic mutations elicit a functional T cell response in the YUMMER 1.7 mouse melanoma model. Pigment Cell Melanoma Res..

[175] Farah C., Neveu M.A., Yelek C., Bouzin C., Gallez B., Baurain J.F., Mignion L., Jordan B.F. (2022). Combined HP ^13^C Pyruvate and ^13^C-Glucose Fluxomic as a Potential Marker of Response to Targeted Therapies in YUMM1.7 Melanoma Xenografts. Biomedicines.

[176] Chitturi Suryaprakash R.T., Shearston K., Farah C.S., Fox S.A., Iqbal M.M., Kadolsky U., Zhong X., Saxena A., Kujan O. (2023). A Novel Preclinical In Vitro 3D Model of Oral Carcinogenesis for Biomarker Discovery and Drug Testing. Int. J. Mol. Sci..

[177] Daneshmandi S., Wegiel B., Seth P. (2019). Blockade of Lactate Dehydrogenase-A (LDH-A) Improves Efficacy of Anti-Programmed Cell Death-1 (PD-1) Therapy in Melanoma. Cancers.

[178] Zhang X. (2022). Isotope tracing reveals distinct substrate preference in murine melanoma subtypes with differing anti-tumor immunity. Cancer Metab..

[179] Yuneva M.O., Fan T.W., Allen T.D., Higashi R.M., Ferraris D.V., Tsukamoto T., Matés J.M., Alonso F.J., Wang C., Seo Y. (2012). The Metabolic Profile of Tumors Depends on Both the Responsible Genetic Lesion and Tissue Type. Cell Metab..

[180] De Feyter H.M., de Graaf R.A. (2021). Deuterium metabolic imaging—Back to the future. J. Magn. Reson..

[181] Polvoy I., Qin H., Flavell R.R., Gordon J., Viswanath P., Sriram R., Ohliger M.A., Wilson D.M. (2021). Deuterium Metabolic Imaging—Rediscovery of a Spectroscopic Tool. Metabolites.

[182] Ouwerkerk R. (2020). Deuterium MR Spectroscopy: A New Way to Image Glycolytic Flux Rates. Radiology.

[183] Ruhm L., Avdievich N., Ziegs T., Nagel A.M., De Feyter H.M., de Graaf R.A., Henning A. (2021). Deuterium metabolic imaging in the human brain at 9.4 Tesla with high spatial and temporal resolution. Neuroimage.

